# Palatability and Swallowability of Pimavanserin When Mixed with Selected Food Vehicles: An Exploratory Open-Label Crossover Study

**DOI:** 10.3390/geriatrics6020061

**Published:** 2021-06-15

**Authors:** Mark Forman, Alex Kouassi, Teresa Brandt, Lee Barsky, Cynthia Zamora, Daryl Dekarske

**Affiliations:** 1 Acadia Pharmaceuticals Inc., San Diego, CA 92130, USA; formanm1240@gmail.com (M.F.); djokourik@gmail.com (A.K.); tbrandt@acadia-pharm.com (T.B.); lee@labadvantage.biz (L.B.); 2 Worldwide Clinical Trials, Morrisville, NC 27709, USA; cynthia.zamora@worldwide.com

**Keywords:** dysphagia, medication administration, Parkinson’s disease, pimavanserin, surveys and questionnaires, swallowing difficulties

## Abstract

Dysphagia (difficulty swallowing) affects up to 13% of persons 65 years and older and 51% of older persons in nursing homes and can contribute to reduced adherence to oral medications. This was an exploratory, single-center, open-label, randomized, crossover study in healthy older adult participants. Primary objectives were evaluation of palatability (taste) and swallowability of the contents of pimavanserin 34 mg capsules mixed with selected soft foods or a liquid nutritional supplement. Secondary objectives included evaluation of additional palatability endpoints and ease of capsule manipulation for mixing. A total of 18 healthy, older adult participants (mean age 65 years) were included. Mean participant ratings for all food vehicles were “moderately like” to “neither like nor dislike” for palatability and “very easy” to “somewhat easy” for swallowability. Capsule manipulation to allow sprinkling of contents was rated “very easy” or “somewhat easy” by most participants. There were five treatment-emergent adverse events, all mild; two were deemed related to study treatment. The palatability and swallowability of pimavanserin was considered acceptable when administered with certain soft foods or a liquid nutritional supplement by the study participants.

## 1. Introduction

Pimavanserin, a selective serotonin receptor-modulating agent with inverse agonist/antagonist activity at 5-HT_2A_ and, to a lesser extent, 5-HT_2C_ receptors [1], is the first US Food and Drug Administration (FDA)-approved treatment for hallucinations and delusions associated with Parkinson’s disease (PD) psychosis [2]. Pimavanserin is available as a powder-filled, 14.4 mm by 5.3 mm immediate-release capsule [3,4]. Older or fragile patients can frequently have difficulty and/or discomfort with swallowing (dysphagia). Dysphagia has been reported to occur in up to 13% of patients over 65 years old, and up to 51% of patients in nursing homes, with increasing prevalence related to age and comorbid conditions [5]. In addition, older adults commonly use multiple medications (polypharmacy) [6], some of which, including neuroleptics, have side effects associated with an increased risk of dysphagia [5,7]. Among patients with PD, dysphagia has an estimated prevalence of 11% to 81% [8] and may be caused by damage to pharyngeal sensory muscles or nerves [9,10]. Dysphagia may cause patients to not comply with oral medication regimens due to anxiety or discomfort regarding swallowing [11].

Administration of oral medications with a small volume of soft foods is a common approach for mitigating issues with swallowing [12]. The FDA has issued draft guidance on the evaluation of soft foods and liquids as food vehicles for administration of oral medications, including considerations for selection of food vehicles and standardized methodology to qualify food vehicles for drug product administration [13]. The palatability of bitter or otherwise unpleasant tasting medications when mixed with foods can be an important factor in compliance and adherence [12]. Nonetheless, there is a lack of accepted methods to evaluate the palatability and swallowability of medications mixed with different food vehicles in clinical settings.

In order to ensure adequate patient acceptability of medications, relevant patient needs across the target patient population should be considered and evaluated. Patient acceptability of medication formulations can be adequately demonstrated by different means, including studies with healthy volunteers [14]. As knowledge on testing patient acceptability in an older population is not well established, the current study employed a customized questionnaire approach to assess the palatability and swallowability of pimavanserin, a medication with a bitter taste [15], when mixed with selected soft foods or a liquid nutritional supplement in healthy older adults. In addition, the ability of the study participants to open and empty pimavanserin capsules for mixing with foods was assessed.

## 2. Materials and Methods

### 2.1. Study Design

**Figure 1:**
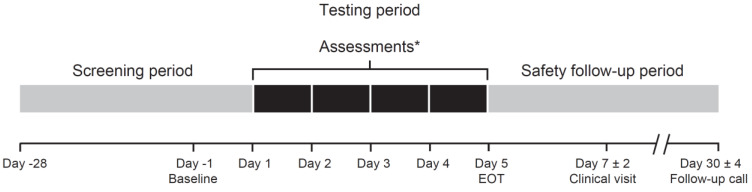
Study design. * Daily testing assessments were divided into 2 parts. In Part 1, participants (*n* = 2) assessed pimavanserin 34 mg mixed with 15 mL or 30 mL food vehicle to determine the minimum acceptable volume. In Part 2, participants (*n* = 16) assessed pimavanserin 34 mg mixed with the minimum volume of food vehicle determined in Part 1. EOT = end of treatment.

This was an exploratory, single-center, open-label, randomized, crossover study consisting of a screening period to assess participant eligibility and collect baseline information, a 5 day treatment period for assessment of pimavanserin food vehicle or liquid nutritional supplement preparations, and a safety follow-up period consisting of a clinic visit and a phone call approximately 7 and 30 days after the last dose of study drug, respectively (Figure 1). Crossover studies allow comparisons between groups in a self-paired fashion, thereby minimizing the risk of inter-participant confounding. As there was no placebo group, neither the study treatment nor the food vehicles were masked (i.e., open-label design).

Based on the recommendations provided by the draft FDA guidance document [13], applesauce, chocolate pudding, and yogurt were selected for assessment as they are readily accessible soft food options among an older population and in long-term care facilities. Vanilla Ensure^®^, a nutritional shake often used as a dietary supplement, was also included as a commonly available liquid nutritional supplement.

Palatability and swallowability assessments were conducted in 2 parts during each treatment day. In Part 1a, 2 participants (1 male and 1 female) assessed pimavanserin mixed with approximately 15 mL (1 tablespoon) of food vehicle for palatability and swallowability to identify the minimum acceptable volume. Based on the draft FDA guidance, palatability was defined as “the quality of a drug product that makes it pleasant or acceptable in terms of taste, aftertaste, smell, and texture,” and swallowability was defined as “the ability of an individual to take the drug without gagging or choking” [13]. If any food vehicle were determined to be unacceptable, it would be evaluated by 2 new participants using approximately 30 mL (2 tablespoons) of food vehicle (Part 1b). An unacceptable rating in Part 1a was defined as a palatability rating of 5 (“dislike extremely”) or a swallowability rating of 4 (“somewhat difficult”) or 5 (“very difficult”) by one or both participants.

Based on the minimum acceptable volume of food vehicle established in Part 1 of the treatment period, each acceptable pimavanserin food vehicle preparation was to be assessed by 16 additional healthy older adult participants in Part 2. As all 4 vehicles were acceptable at 15 mL, Part 2 of this study used a 4-way Latin square crossover design with 4 treatments (food vehicles) in 15 mL volume, 4 periods, and 4 sequences balanced for first-order carryover effects. Study participants were randomly assigned to 1 of the 4 sequences in a 1:1:1:1 ratio using permuted blocks on study day 1. The randomization was stratified by sex. Secondary endpoints were assessed during each of the 5 study period days. The ease of capsule manipulation was assessed during Part 2 of each treatment day.

This study was conducted in compliance with the protocol, the Declaration of Helsinki, International Council for Harmonisation Good Clinical Practice, and other applicable regulatory requirements. The study protocol was approved by the institutional review board, and all participants provided informed written consent.

### 2.2. Participant Selection

Healthy, male and female adults ≥ 60 years of age were eligible for participation in this study in order to best represent a generally older PD patient population. A determination of good health was judged by the investigator based on screening medical history, physical examination, laboratory test profile, vital signs, and electrocardiogram. Any stable or chronic disease must have been judged by the investigator as stable and unlikely to interfere with the ability of the individual to participate in this study. Individuals with illnesses, including the common cold, dysgeusia, or active seasonal allergies, or those taking a medication that could alter sense of taste, were excluded.

### 2.3. Treatment Administration

The investigational product administered with the food vehicles was the commercially available pimavanserin 34 mg immediate-release capsule, which contains white to off-white powders. The capsule is 14.4 mm by 5.3 mm, and made of 2 separable capsule components (1 white, 1 green) [4]. Study participants stayed overnight in the clinic until the study procedures were completed. Participants fasted overnight prior to dosing and did not eat for 1 h afterwards. In the morning of treatment days 1–4, participants received the contents of 1 pimavanserin 34 mg capsule mixed into 1 of the 4 food vehicles (applesauce, chocolate pudding, yogurt, or vanilla Ensure^®^) by a study administrator. Participants were instructed to flush their mouth with 250 mL of water immediately before and immediately after treatment administration. Assessments of palatability and swallowability were made immediately after flushing with water. Pimavanserin was mixed with a different food vehicle for assessment on days 1–4 of the treatment period.

### 2.4. Endpoints and Assessments

Primary study endpoints were the assessment of palatability (taste) and swallowability pimavanserin when mixed with selected food vehicles. Five-point (1 to 5) hedonic scales were used to rate the taste dimension of palatability (like extremely, like moderately, neither like nor dislike, dislike moderately, dislike extremely) and swallowability (very easy, somewhat easy, neither easy nor difficult, somewhat difficult, very difficult) of pimavanserin food vehicle or liquid nutritional supplement preparations. Across rating scales, a lower score represents a more favorable rating (Supplementary Materials, Table S1).

Secondary endpoints included ratings of additional palatability measures (mouthfeel [texture], smell, aftertaste, and bitterness), all on a 1- to 5-point scale (Table S1). Aftertaste was assessed at the end of treatment (nominally day 5) at 1, 5, and 15 min and 1, 8, and 24 h after dosing. Primary and secondary endpoints for palatability and swallowability were assessed during Part 1a and Part 2 of each treatment day. An additional secondary endpoint, ease of capsule manipulation (opening and emptying capsule contents into food) was assessed during Part 2, also using a 1- to 5-point scale (Table S1). This endpoint was assessed separately from the secondary endpoints for palatability and swallowability. The ease of capsule manipulation by study participants was also evaluated by an observing site staff member who provided a yes or no answer to the following question: “Was the participant able to independently open the capsule and empty its contents?”

Safety was assessed by monitoring for treatment-emergent adverse events (TEAEs) and potential changes in clinical evaluations. Safety laboratory evaluations were performed at screening, baseline, end of study (or early termination), and the follow-up safety visit.

### 2.5. Statistical Analyses

This was an exploratory study and was not powered to achieve statistical significance. Study outcomes are summarized descriptively. For all assessments of palatability (including taste, mouthfeel, smell, and aftertaste), a favorable response was defined as a rating of 1–3 (like extremely, like moderately, neither like nor dislike); an unfavorable response was defined as a rating of 4 or 5 (dislike moderately, dislike extremely). For swallowability, assessments of 1–3 (very easy, somewhat easy, neither easy nor difficult) were defined as favorable, and scores of 4 or 5 (somewhat difficult, very difficult) defined as unfavorable. For assessment of bitterness, a favorable response was defined as a score of 1 or 2 (no bitterness, mild bitterness); unfavorable responses were defined as scores of 3–5 (moderate bitterness, severe bitterness, extreme bitterness). Similarly, a rating of 1–3 for capsule manipulation (very easy, somewhat easy, neither easy nor difficult) was defined as favorable and ratings of 4–5 (somewhat difficult, very difficult) as unfavorable.

Analyses of primary and secondary endpoints were performed on the full analysis set (FAS), which included all randomized and treated participants who had at least one rating score assessing the palatability or swallowability of a pimavanserin food vehicle preparation. Safety analyses were completed on the safety analysis set (SAS), which included all participants who received at least one dose of pimavanserin.

## 3. Results

### 3.1. Study Population

This study was conducted at a single site in the United States from 14 January 2020 to 2 March 2020. Two healthy older adults participated in Part 1a of this study and 16 healthy older adults were involved in Part 2 of this study, for a total of 18 participants (Table 1). All of the 18 participants completed this study, each receiving all 4 pimavanserin food vehicle and liquid nutritional supplement preparations. Therefore, the FAS and SAS were identical. At baseline, participants had a mean (standard deviation [SD]) age of 65.2 (4.9) years, and 9 of 18 (50%) were female. (Table 1).

### 3.2. Food Vehicle Volume Assessments

Two participants, 1 male and 1 female, were included in Part 1a (small volume testing) on each treatment day. During Part 1a, no pimavanserin/food vehicle or liquid nutritional supplement mixture received a rating of unacceptable for palatability or swallowability from either participant (Table S2). Therefore, Part 1b (large volume testing) was not performed and Part 2 was conducted with the small volume (approximately 15 mL) for all food vehicles and liquid nutritional supplement. The results from the 2 participants in Part 1a are included in the FAS.

### 3.3. Primary Study Endpoints

#### Palatability (Taste) and Swallowability

**Figure 2:**
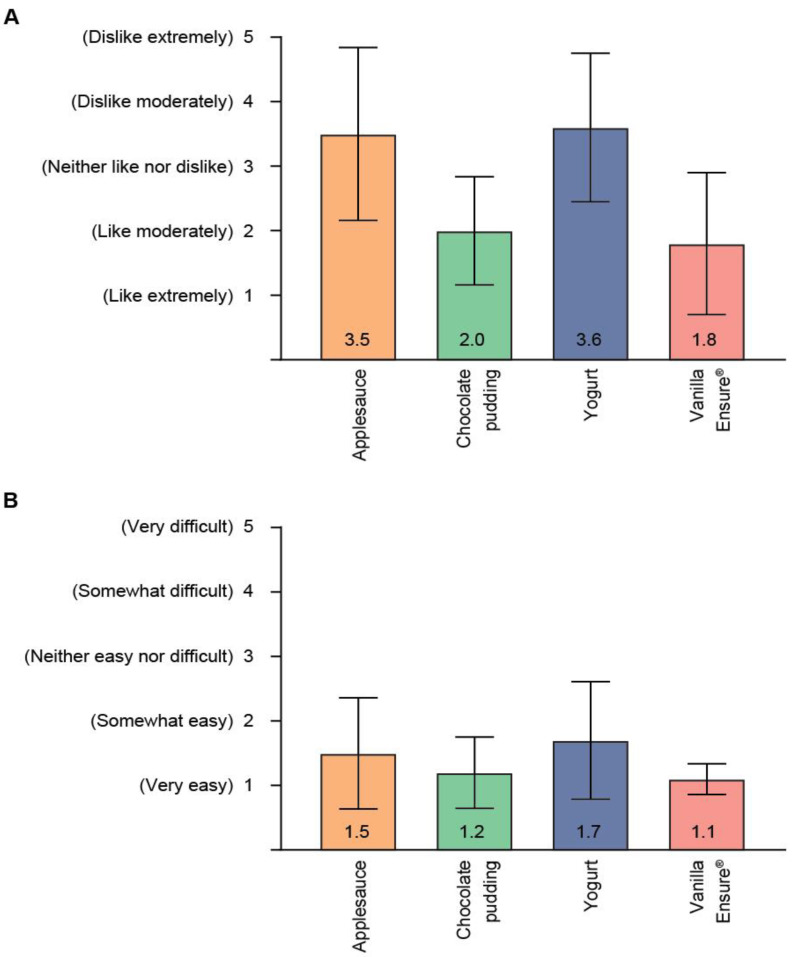
Assessments of (**A**) palatability and (**B**) swallowability of pimavanserin/food vehicle mixtures. Mean (SD) participant assessments (*n* = 18) of pimavanserin 34 mg mixed with 15 mL of indicated food vehicle. SD = standard deviation.

For all pimavanserin mixtures tested, the mean (SD) ratings for taste ranged from 1.8 (1.10) for vanilla Ensure^®^ to 3.6 (1.15) for yogurt (Figure 2A). Favorable assessments for taste (score 1–3) were provided by 17 of 18 (94%) of study participants for chocolate pudding, 15 of 18 (83%) for vanilla Ensure^®^, and 6 of 18 (33%) each for applesauce and yogurt. Of the 12 participants who did not rate the taste for plain yogurt and applesauce as favorable, 8 and 9 participants, respectively, assessed it as dislike moderately. Overall, 6 participants gave a rating of 5 (dislike extremely) for at least 1 food vehicle: 2 for plain yogurt, 3 for applesauce, and 1 each for yogurt and applesauce. It should be noted, none of these participants considered swallowability very difficult (see below).

The mean (SD) swallowability ratings ranged from 1.1 (0.24) for vanilla Ensure^®^ to 1.7 (0.91) for yogurt (Figure 2B). All 18 study participants gave favorable responses (score 1–3) for swallowability of applesauce, chocolate pudding, and vanilla Ensure^®^. Seventeen of 18 (94%) participants gave a favorable response for yogurt. There were no swallowability scores of 5 (very difficult) for any pimavanserin mixtures, and only 1 food vehicle (yogurt) received a rating of 4 (somewhat difficult) by 1 of 18 (6%) participants.

There were no notable differences in palatability (taste) or swallowability assessments between study days or subgroups stratified by sex.

### 3.4. Secondary Study Endpoints

#### 3.4.1. Mouthfeel, Smell, Aftertaste, and Bitterness

**Figure 3:**
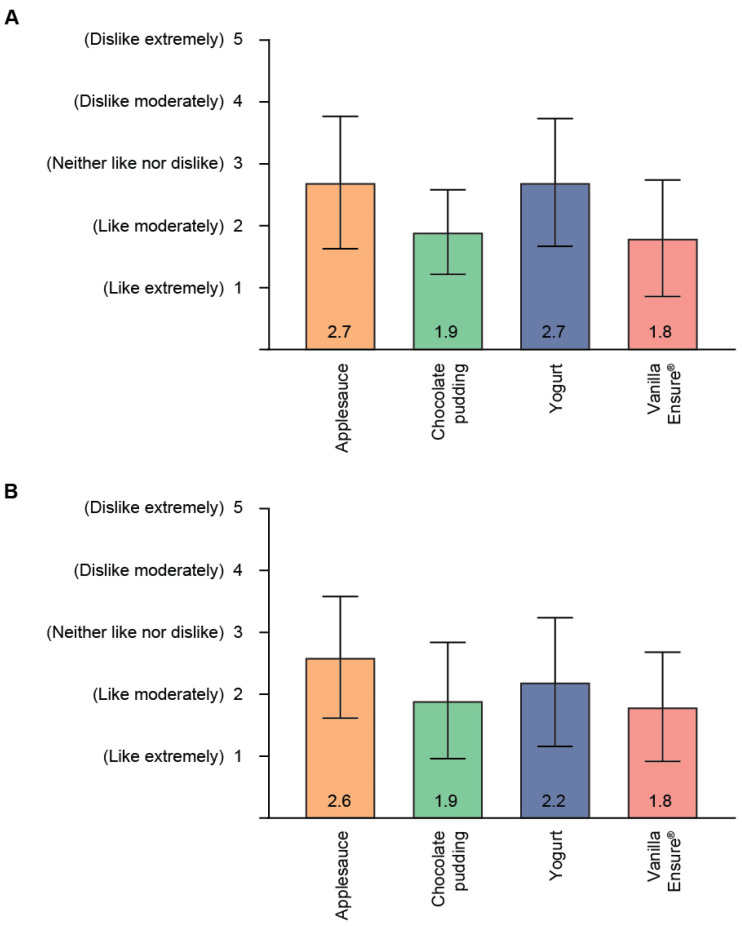

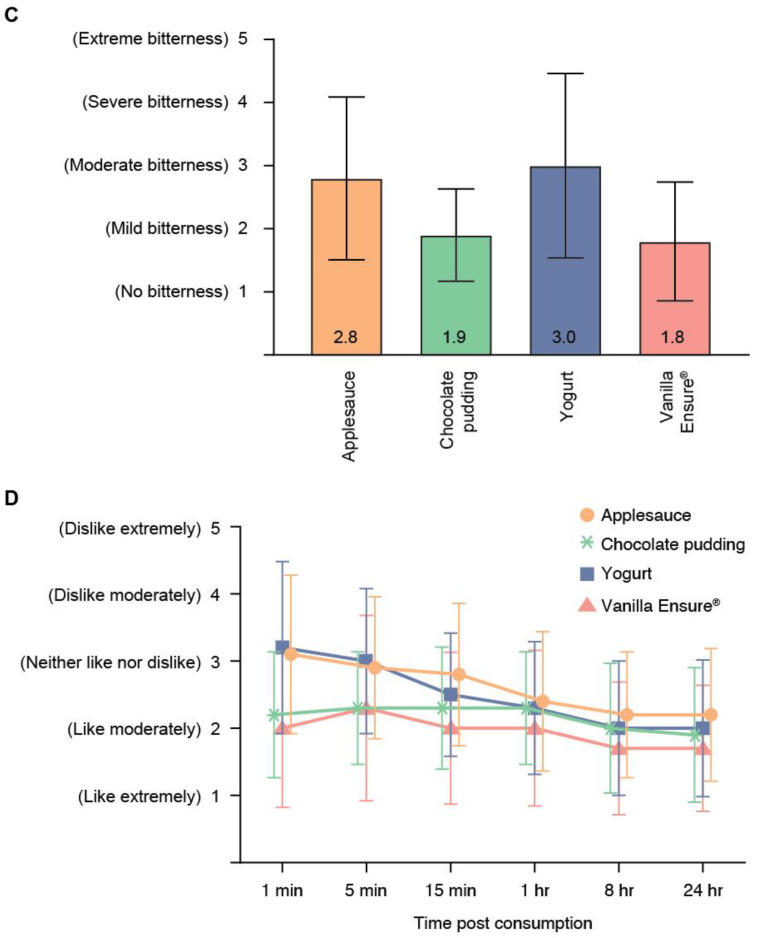
Secondary endpoint assessments of (**A**) mouthfeel, (**B**) smell, (**C**) bitterness, and (**D**) aftertaste of pimavanserin/food vehicle mixtures. Data shown are the mean (SD) of participant assessments (*n* = 18) of pimavanserin 34 mg mixed with 15 mL of indicated food vehicle. SD = standard deviation.

The ratings for the additional palatability measures of mouthfeel, smell, bitterness, and aftertaste were generally favorable (Figure 3A–D). As shown in Figure 3A, the majority of study participants provided favorable responses for mouthfeel across all pimavanserin mixtures (18 participants [100%] for chocolate pudding and vanilla Ensure^®^, 15 participants [83%] for yogurt, and 14 participants [78%] for applesauce). There were only 2 mouthfeel scores of 5 (dislike extremely) in 2 participants (1 each for yogurt and applesauce). Mean scores for smell are depicted in Figure 3B. A majority of study participants gave favorable responses for smell for all pimavanserin mixtures (18 participants [100%] for chocolate pudding and vanilla Ensure^®^, 17 participants [94%] for yogurt, and 16 participants [89%] for applesauce). There were no scores of 5 (dislike extremely) for smell.

Mean bitterness scores are shown in Figure 3C. The bitterness of pimavanserin mixed with chocolate pudding or vanilla Ensure^®^ was rated as favorable (no or mild bitterness) by 14 (78%) of participants, while 9 (50%) and 8 (44%) of participants rated the bitterness of pimavanserin mixed with applesauce and yogurt, respectively, as favorable. Six participants assessed the bitterness for at least 1 pimavanserin mixture as 5 (extreme bitterness): 3 participants for yogurt, 2 participants for applesauce, and 1 participant for both yogurt and applesauce.

Mean scores for aftertaste, assessed at end of treatment on day 5, of the different pimavanserin mixtures are provided in Figure 3D. One-minute post consumption, the majority of study participants provided favorable responses for aftertaste: 17 participants (94%) for chocolate pudding, 15 participants (83%) for vanilla Ensure^®^, 11 participants (61%) for yogurt, and 10 participants (56%) for applesauce (Figure S1). The number of participants giving favorable responses for aftertaste increased to 100% by 1-h post consumption for pimavanserin mixed with chocolate pudding or vanilla Ensure^®^, and by 8 h post consumption for pimavanserin mixed yogurt or applesauce. Between 1 and 15 min post consumption, 5 participants gave aftertaste scores of 5 (dislike extremely): 2 for yogurt, 2 for vanilla Ensure^®^, and 1 for both yogurt and applesauce (Figure S1).

There were no notable differences in any of the secondary endpoint assessments between study days or subgroups stratified by sex.

#### 3.4.2. Capsule Manipulation

**Figure 4:**
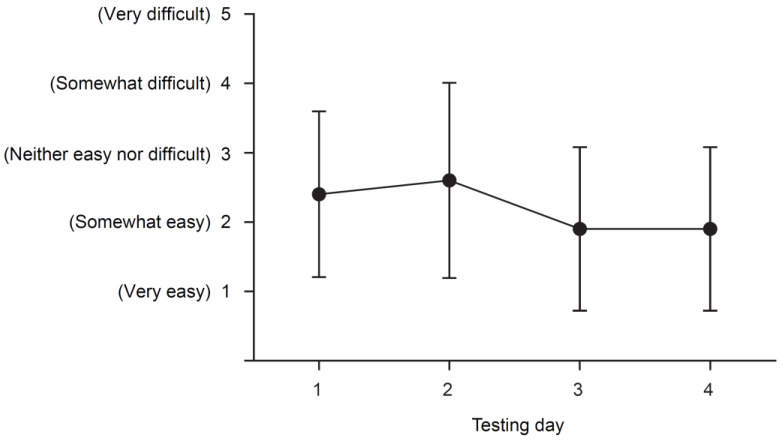
Assessment of ease of pimavanserin capsule manipulation. Data shown are the mean (SD) of participant assessments (*n* = 16) of ease of capsule manipulation, defined as opening and emptying capsule contents. SD = standard deviation.

Scores for ease of opening and emptying the pimavanserin 34 mg immediate-release capsule ranged from 2.6 (SD 1.41) to 1.9 (SD 1.18) over the 4 assessments (Figure 4). The site staff found that at least 14 participants (88%) on each day were able to open the capsule and empty the contents. The majority of participants provided favorable responses on each study day, with 11/18 participants (69%) responding favorably on days 1 and 2 and 13/18 participants (81%) responding favorably on days 3 and 4.

### 3.5. Safety

During this study, TEAEs were reported by 5 of 28 (28%) participants (Table 2). Two participants (11.1%) experienced TEAEs that were judged by the study investigator to be related to study treatment (diarrhea and vomiting). All TEAEs were mild and there were no serious TEAEs, TEAEs leading to discontinuation, or TEAEs resulting in death. There were no clinically significant changes in laboratory values and no clinically significant findings from physical assessments or measures of vital signs.

## 4. Discussion

Among the healthy, older participants tested, pimavanserin mixed with certain types of soft foods or a liquid nutritional supplement generally received favorable ratings on measures of palatability and swallowability. Taste was rated favorably by most participants for chocolate pudding and vanilla Ensure^®^ and by one-third of participants each for applesauce and yogurt. Across all food vehicles, almost all participants gave favorable responses for swallowability (99%) and mouthfeel (90%), and by a majority of participants for aftertaste, recorded 1 min post consumption (74%). Only 15 mL of each food vehicle or liquid nutritional supplement was necessary for participant acceptance.

On all study days, the majority of participants gave favorable responses for capsule manipulation, rating ease of capsule manipulation as either neutral or positive, and site staff judged that the majority of participants were able to open the capsule and empty the contents on each day. TEAEs experienced by the study participants were mild and only two TEAEs were judged by the investigator to be related to the study treatment. There were no deaths, serious TEAEs, or TEAEs leading to withdrawal.

Oral administration of medication is often preferred by patients based on its convenience and acceptability [16,17,18]. However, adherence to oral medications may be hindered by swallowing difficulties [17,18]. Dysphagia is common, occurring in 7% to 13% of individuals over 65 years of age and over 50% among those in nursing homes [19]. The prevalence for patients with PD is higher, ranging from 11% to 81% [8] and is typically most severe with advanced PD [20].

Regulatory guidance on the use of food vehicles for administration of medications is in its early stages and evaluation scales are in earlier stages of development and testing. Thus, there remains a lack of standardized scales and assessments for the quantification of palatability and swallowability. In a recent systematic review, the most common approach for quantifying patient acceptance was a questionnaire; however, the studies identified lacked consistent criteria for what constituted an acceptable result [12]. Furthermore, the determination of acceptability was subjective and not based on a priori statistical comparisons, thus the acceptability studies frequently did not have a control group. In studies of healthy adult participants similar to this one, simple, 5-point hedonic scales were used for participants to rate their experience across a variety of dimensions [21,22,23].

Despite their potential inherent limitations, simple rating scales are a commonly accepted method for evaluating the use of food vehicles and were therefore chosen for the current study. The rating scales adapted and developed for this study demonstrated consistent results over multiple days for multiple assessments. The study size, while appropriate for an exploratory study, precluded analyses for statistical significance. The consistency of the results presented here combined with the composition of the study population should assist in generalizability of the findings to elderly and sometimes frail patients with PD and PD psychosis. As the pathology of dysphagia and related disorders is complex, additional studies in patients with these swallowing disorders would be informative.

The results of this study in healthy older adult participants demonstrate that pimavanserin capsules are easy to open and that the capsule contents are palatable and easy to swallow when mixed with applesauce, yogurt, pudding, or a liquid nutritional supplement. The results of this study may additionally help provide guidance for patients who are taking pimavanserin, but face difficulties with swallowing [3].

## Figures and Tables

**Table 1:** Baseline characteristics of study participants.

Characteristic	Overall (*n* = 18)
Sex, *n* (%)	
Female	9 (50.0)
Male	9 (50.0)
Age, mean (SD)	65.2 (4.9)
Race, *n* (%)	
American Indian or Alaskan Native	1 (5.6)
Asian	1 (5.6)
Black or African American	1 (5.6)
White	15 (83.3)
Ethnicity	
Hispanic or Latino	8 (44.4)
Not Hispanic or Latino	10 (55.6)
BMI, mean (SD), kg/m^2^	26.1 (3.7)

BMI = body mass index; SD = standard deviation.

**Table 2:** Treatment-emergent adverse events in the full analysis set.

TEAE, *n* (%) ^a^	Overall (*n* = 18)
Any TEAE	5 (27.8)
Gastrointestinal disorders	4 (22.2)
Constipation	1 (5.6)
Diarrhea	1 (5.6) ^b^
Lip dry	1 (5.6)
Vomiting	1 (5.6) ^b^
Musculoskeletal and connective tissue disorders	1 (5.6)
Back pain	1 (5.6)

^a^ TEAEs listed by Medical Dictionary for Regulatory Activities (MedDRA; ver. 22.1) System Organ Class and Preferred Term. Participants are counted once within each System Organ Class and Preferred Term. ^b^ TEAE related to study treatment by the investigator. TEAE—treatment-emergent adverse event.

## Data Availability

The data presented in this publication are not publicly available. For access, please contact the corresponding author with request details.

## Supplementary Materials

The following are available online at https://www.mdpi.com/article/10.3390/geriatrics6020061/s1, Table S1: Five-point scales used for primary and secondary endpoint assessments; Table S2: Palatability and swallowability assessments of pimavanserin/food vehicle mixtures from Part 1a (minimum food volume testing); Figure S1: Percentage of participants with favorable response for aftertaste of pimavanserin/food vehicle mixtures over time
