# Supplementary material for: Palatability and Swallowability of Pimavanserin When Mixed with Selected Food Vehicles: An Exploratory Open-Label Crossover Study
Source: Geriatrics (Basel). 2021 Jun 15;6(2):61. doi: 10.3390/geriatrics6020061 (PMC8293179; doi:10.3390/geriatrics6020061)
Supplement: Supplementary file 1 [file geriatrics-06-00061-s001.zip › geriatrics-1200719-supplementary.pdf]

Supplementary Material

Table S1. Five-point scales used for primary and secondary endpoint assessments

	Primary Endpoints			Secondary Endpoints			
**Rating**	**Taste (palatability)**	**Swallowability**	**Mouthfeel (texture)**	**Smell**	**Bitterness**	**Aftertaste**	**Capsule manipulation (participant assessment)**
1	Like extremely	Very easy	Like extremely	Like extremely	No bitterness	Like extremely	Very easy
2	Like moderately	Somewhat easy	Like moderately	Like moderately	Mild bitterness	Like moderately	Somewhat easy
3	Neither like nor dislike	Neither easy nor difficult	Neither like nor dislike	Neither like nor dislike	Moderate bitterness	Neither like nor dislike	Neither easy nor difficult
4	Dislike moderately	Somewhat difficult	Dislike moderately	Dislike moderately	Severe bitterness	Dislike moderately	Somewhat difficult
5	Dislike extremely	Very difficult	Dislike extremely	Dislike extremely	Extreme bitterness	Dislike extremely	Very difficult

https://doi.org/10.3390/geriatrics6020061

Table S2. Palatability and swallowability assessments of pimavanserin/food vehicle mixtures from Part 1a (minimum food volume testing)

	Palatability				Swallowability			
**Assessment**	**Applesauce**	**Chocolate Pudding**	**Yogurt**	**Vanilla Ensure®**	**Applesauce**	**Chocolate Pudding**	**Yogurt**	**Vanilla Ensure®**
Participant 1	4<br>(dislike moderately)	1<br>(like extremely)	2<br>(like moderately)	4<br>(dislike moderately)	1<br>(very easy)	1<br>(very easy)	1<br>(very easy)	1<br>(very easy)
Participant 2	2<br>(like moderately)	2<br>(like moderately)	4<br>(dislike moderately)	2<br>(like moderately)	1<br>(very easy)	2<br>(somewhat easy)	2<br>(somewhat easy)	2<br>(somewhat easy)

For Part 1a assessments, pimavanserin was mixed with 15 mL (1 tablespoon) of food vehicle.
Palatability rating scores were 1 (like extremely), 2 (like moderately), 3 (neither like nor dislike), 4 (dislike moderately), 5 (dislike extremely).
Swallowability rating scores were 1 (very easy), 2 (somewhat easy), 3 (neither easy nor difficult), 4 (somewhat difficult), 5 (very difficult).

Figure S1. Percentage of participants with favorable response for aftertaste of pimavanserin/food vehicle mixtures over time

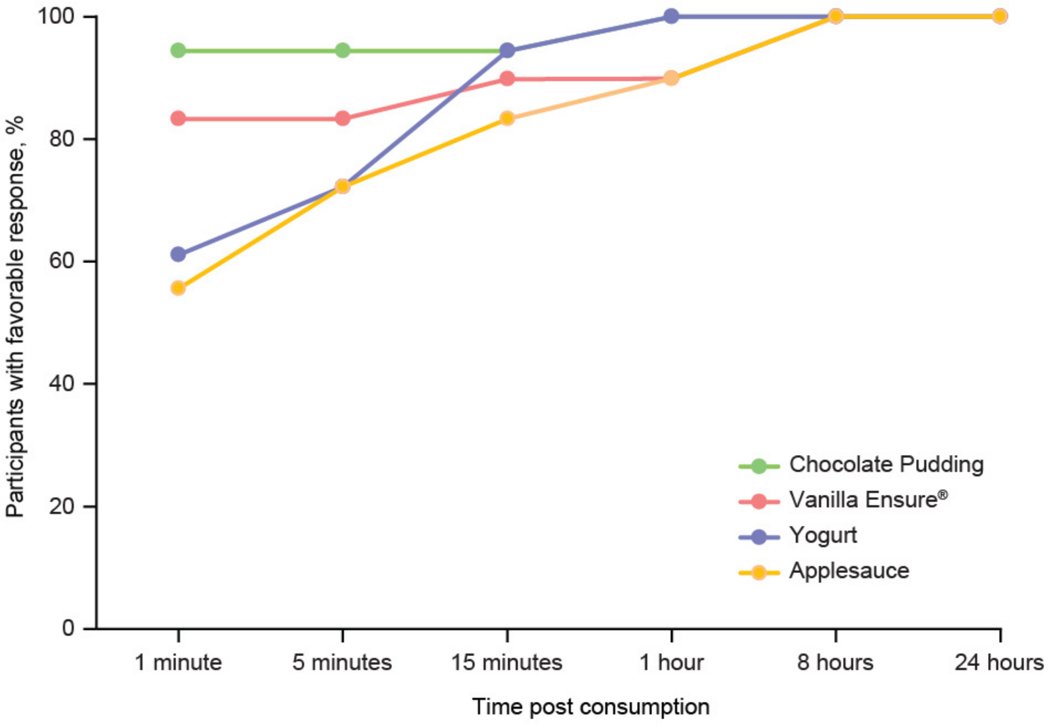

A favorable response for aftertaste was defined as a score of 1–3 (like extremely, like moderately, neither like nor dislike).

https://doi.org/10.3390/geriatrics6020061
